# Confronting the Colonial Roots of Global Health Inequities in Gaza: Comment on "The Rhetoric of Decolonizing Global Health Fails to Address the Reality of Settler Colonialism: Gaza as a Case in Point"

**DOI:** 10.34172/ijhpm.8768

**Published:** 2024-10-28

**Authors:** Guido Veronese, Ashraf Kagee, Yasser Abu Jamei

**Affiliations:** ^1^Department of Human Sciences and education “R. Massa”, Università degli Studi di Milano-Bicocca, Milano, Italy.; ^2^Department of Psychology, Stellenbosch University, Stellenbosch, South Africa.; ^3^Gaza Community Mental Health Program, Gaza, Palestine.

**Keywords:** Colonialism, Global Health, Economic Extractivism, Health Subalternity, Gaza Genocide

## Abstract

This response critically examines the editorial by Engebretsen and Baker, emphasizing the colonial underpinnings of global health as it pertains to Gaza. We argue that global health is not merely ineffective but complicit in perpetuating settler colonial structures that exacerbate health disparities. The health crisis in Gaza is intricately linked to Israeli occupation, challenging the reductionist frames of "conflict health" and "refugee health" often employed by global health institutions. The presence of non-governmental organizations (NGOs) in Gaza exemplifies how international health efforts can depoliticize the crisis, as they often operate within constraints that do not challenge the underlying power dynamics. Our call for localization and self-determination highlights the complexities of achieving these goals in a context where the state is unrecognized. To effect meaningful change, global health must confront and dismantle the colonial structures underpinning health inequities in Gaza, moving beyond superficial humanitarian approaches to advocate for justice and autonomy.

 The editorial by Engebretsen and Baker rightly addresses the colonial underpinnings of global health but stops short of unapologetically acknowledging the colonial nature of global health as part of an oppressive system, rather than as a disembodied discipline that is strong in theory but weak in action.^1^ Our response emphasizes that global health, as currently practiced, is not merely ineffective but complicit in maintaining settler colonial structures, particularly in Gaza, where health inequities are deeply intertwined with the ongoing occupation.

## The Colonial Nature of Global Health in Gaza

 The editorial critiques global health’s theoretical limitations but does not fully engage with the reality that global health, as it operates in Gaza, is complicit in settler colonial policies. The health crisis in Gaza cannot be reduced to a “conflict health” or “refugee health” issue as often framed by global health institutions.^2^ Instead, it is directly tied to Israeli settler colonialism, which systematically undermines Palestinian health systems. This framework, adopted by many global health institutions, national and supranational non-governmental organizations (NGOs), obscures the true cause of poor health outcomes: the occupation and siege, creating conditions where Gaza is treated as a passive site for intervention, rather than a place where health systems are systematically dismantled by colonial power.^3^

 After October 7th, Richard Horton, editor-in-chief of *The Lancet*, published an editorial on Gaza that starkly reflects the racist underpinnings of global health’s treatment of the region. In his editorial, Horton not only accused Hamas but also, by extension, the people of Gaza of barbarism—reinforcing harmful colonial stereotypes of Palestinians as backwards or uncivilized.^4^ This framing perpetuates the very colonial logic that global health institutions must confront, presenting Palestinians not as victims of an unjust occupation and ethnic cleansing, but as participants in their own suffering. By doing so, Horton’s discourse feeds into the broader narrative of dehumanization that justifies ongoing violence against Gaza and erases the colonial structures actively oppressing the population. His editorial, emblematic of a racist narrative, reduces Gaza’s health crisis to an issue of internal fault rather than recognizing the external forces—namely settler colonialism and occupation—that systematically dismantle its health infrastructures.^5,6^

## NGOization and Its Unique Impact in Gaza

 The NGOization of healthcare in Gaza is a prime example of how global health’s colonial nature manifests. While the global proliferation of NGOs is widely critiqued, in Gaza, the dynamic is uniquely shaped by the occupation. International NGOs in Gaza are not merely filling gaps in the health system, but often become complicit in depoliticizing the Palestinian health crisis by operating within Israeli-imposed restrictions.^7^ Unlike in Haiti, which has the highest per capita rate of NGOs in the world, the presence of NGOs in Gaza is tightly controlled. NGOs are often granted permission to operate only under conditions that do not challenge the Israeli occupation, reflecting what Bhungalia describes as an “elastic empire”^5^—an imperial reach that adjusts itself to maintain dominance, while shifting the burden of care to international agencies without addressing the structural issues causing the health crisis.

## Localization and Self-determination: Complex Realities

 Our call for localization and self-determination in the Palestinian health sector requires a deeper exploration of the complexities on the ground. Localization and self-determination, while important, have themselves been co-opted by liberal global health reforms. In Gaza, a region where the state is not recognized by many powerful actors, the notion of self-determination is difficult to realize. Localization often becomes a tool for managing crises without addressing their root causes—Israeli occupation and settler colonialism.^8^ True self-determination in Gaza’s health sector would require dismantling these structures, not merely localizing or decentralizing existing frameworks of global health, which are often complicit in sustaining the occupation.

## International Law and Its Limitations in Global Health

 The editorial rightfully critiques the limitations of international law in addressing health inequities in Gaza. While we advocate for global health interventions that adhere to international laws rather than Israeli regulations, we must acknowledge that international law itself has often failed Palestinians. As the editorial notes, institutions like the World Health Organization (WHO) are deeply entangled in settler colonial logics, reducing Gaza’s health crisis to a humanitarian issue while sidestepping the political causes. By framing Gaza’s health crisis as a question of conflict health or humanitarian assistance, international law and global health institutions obscure the political causes of poor health outcomes in Gaza, making it nearly impossible to enact meaningful change.^9^ To challenge this, global health must recognize the colonial structures that underpin its frameworks and actively work to dismantle them, rather than applying band-aid solutions to a deeply rooted problem.^10^

## Fanon and the Coloniality of Medicine

 Our invocation of Fanon aligns with his critique of colonial medicine,^11^ but we acknowledge that Fanon’s views on Western medicine were more nuanced. Fanon did not reject Western medicine outright; rather, he argued that medicine in a colonial context could never be neutral. His critique of colonial medicine was rooted in the understanding that medical institutions, even when led by non-Western actors, were often entangled in the broader colonial system. In Gaza, the destruction of hospitals and medical infrastructure reflects this reality.^12^ Israel’s attacks on hospitals and health centers are not simply an act of viewing Palestinian health institutions as inferior but are part of a broader strategy to disable Palestinian society as a whole. As Puar^13^ argues, this tactic of biopolitical control is designed to render the Palestinian population dependent and ungovernable.

## Conclusion

 In conclusion, we argue that the failure of global health in Gaza is not just one of inefficacy but of complicity in settler colonialism. By framing Gaza’s health crisis as a humanitarian issue, global health institutions obscure the true causes and reinforce the status quo.^14^ To decolonize global health, we must confront the settler colonial structures that underpin the health inequities in Gaza and move beyond depoliticized frameworks that reduce the Palestinian health crisis to a matter of charity, rather than justice.^15^ The ongoing global student protests represent a strong call for change, drawing attention to the scholsticide,^16^ the destruction of civilian infrastructure, and the erasure of Gaza’s health system. These demonstrations highlight the urgent need to examine the colonial roots of global health and push for reforms that go beyond superficial measures. As the editorial notes, Gaza’s health crisis is not merely a medical issue; it reflects a deeper reality of colonial oppression. To effectively address health inequities in Gaza, global health must engage critically with these intertwined challenges.

 The root cause of the health crisis is fact that Palestinians were dispossessed of their land in 1948. The consequent political and military crimes such as occupation, siege, and ongoing suppression of dissent against Zionism have been perpetrated to uphold and maintain the dispossession of Palestinian land, the denial of Palestinian political identity and the continued violation of political rights. If this root cause is not addressed, there is little likelihood of global health having any significant impact on healthcare in Gaza. Such a radical rethinking is necessary, of acknowledging the crime of the Nakba, ie, ethnic cleansing, land theft, and political annihilation both in 1948 but also in its present form of genocide, culturecide, epistemicide, and scholasticide. An action-to-knowledge strategy therefore challenges the moral and ethical foundations of Zionism and seeks to create a just peace, characterised by human rights for all, not only for Jews. Such a vision provides some direction for Engebretsen and Baker’s proposal to radically rethink the position of global health institutions. In this sense, we see neoliberalism, capitalism, settler-colonialism and Zionism as interrelated. Systems of knowledge production therefore need to challenge these assumption at their very foundation

## Ethical issues

 Not applicable.

## Conflicts of interest

 Authors declare that they have no conflicts of interest.
